# Cultural Triggers of Dissociative Fugue: From Workplace Stress to Early Life Adversity in a South Asian Male

**DOI:** 10.1192/j.eurpsy.2026.11463

**Published:** 2026-07-31

**Authors:** A. Arshad, A. Khalid, M. Bilal, I. Arshad

**Affiliations:** 1 Medicine, Pakistan Medical and Dental Council, Gujranwala, Pakistan; 2 Internal Medicine, Aiken Regional Medical Centers, Aiken, South Carolina, United States; 3 Psychiatry, Allama Iqbal Medical College, UHS, Lahore; 4 Internal Medicine, Gujranwala Teaching Hospital, Gujranwala, Pakistan

## Abstract

**Introduction:**

Dissociative fugue is a rare condition, falling within the spectrum of dissociative identity disorders as per the DSM-V criteria. This condition is marked by an abrupt and unforeseen departure from one’s usual routine, accompanied by memory lapses. Its occurrence within the general population is estimated at a mere 0.2%, and individuals may linger in this fugue state for a few days to several months. Typically, the onset of a fugue episode is sudden and often linked to traumatic or highly stressful life events

While often linked to acute stress its presentation in existing literature on dissociative fugue is sparsely documented within the south Asian populations.

**Objectives:**

This case report aims to highlight the psychosocial and cultural triggers of dissociative fugue by detailing a presentation in a South Asian male.

**Methods:**

A 35 year old man from Pakistan who presented after a 5-day wandering episode associated with disorientation and an inability to recognize familiar faces. Identified risk factors included workplace stress, financial pressures, and a history of parental estrangement. Mental examination revealed disorientation to place and anxiety. Medical workup, including CBC, LFTs, RFTs, CT scan, and EEG, was unremarkable, ruling out neurological disorders ;however, the presentation along with the dissociative experience scale score of 50 (clinical cutoff ≥ 30) supported the diagnosis of Dissociative Fugue.

**Results:**

Management involved psychotherapy and coping strategies, coupled with medication for depression and anxiety. The patient showed improvement with the management and showed partial recovery of memories and improved daily functioning.

**Conclusions:**

This case report demonstrates the importance of considering dissociative disorders in the differential diagnosis of individuals who present with unexplained amnesia and fugue states. A comprehensive assessment, including the standardized tools such as DES, is important. Early intervention, multidisciplinary approach and awareness about the triggers which can cause such episodes including cultural and workplace stressors and childhood adverse effects is essential for improving outcomes in dissociative disorders.

**Disclosure of Interest:**

None Declared

